# Prosociality predicts connectedness with nature and soil

**DOI:** 10.3389/fpsyg.2026.1803464

**Published:** 2026-05-01

**Authors:** Alexander Neaman, Gonzalo Palomo-Vélez, Mónica Castro, P. Wesley Schultz

**Affiliations:** 1 Facultad de Ciencias Agronómicas, Universidad de Tarapacá, Arica, Chile; 2 Faculty of Behavioral and Social Sciences, University of Groningen, Groningen, Netherlands; 3 Escuela de Agronomía, Pontificia Universidad Católica de Valparaíso, Quillota, Chile; 4 Claremont Evaluation Center, Claremont Graduate University, Claremont, CA, United States

**Keywords:** agriculture, altruism, nature connectedness, prosocial propensity, soil connectedness, soil conservation

## Abstract

Prosocial propensity refers to an individual’s general inclination to act for the benefit of others. Notably, psychological research has often defined “others” narrowly, limiting the term to humans. However, some authors argue that the concept of “others” should be expanded to include the natural word. Previous research has demonstrated that an individual’s prosocial propensity underpins their connectedness to nature (also referred to as “connectedness with nature” or “nature relatedness”). Accordingly, the main objective of this study was to examine the relationship between prosocial propensity and soil connectedness in a sample of Chilean farmers (*n* = 166). Altruism was used as an indicator of prosocial propensity to examine its association with a newly developed Inclusion of Soil in Self measure, inspired by the well-known Inclusion of Nature in Self (INS) measure, thereby assessing the relationship between prosocial tendencies and connectedness to soil. First, a Rasch model was used to compute scores for the altruism scale. The Rasch residuals explained 10% of the variance, suggesting that the scale adequately captured a single underlying construct. Then, correlation analyses revealed that prosocial propensity was positively associated with connectedness to both nature (*r* = 0.22, *p* < 0.01) and soil (*r* = 0.24, *p* < 0.01). These associations remained robust when controlling for income, education, age, and gender. This study contributes to the growing body of evidence that environmentalism is an indicator of prosociality, and that connectedness to nature and soil may reflect a domain in which prosocial tendencies are expressed.

## Introduction

Prosocial propensity refers to the tendency to help others (Angelis and Pensini, 2023). From a behavioral perspective, prosocial tendencies have commonly been conceptualized in terms of altruism, defined as behavior that benefits others at some cost to the actor (Batson and Powell, 2003). Importantly, however, the fact that altruistic behavior benefits others at a personal cost does not necessarily imply that such behaviors are entirely selfless. Indeed, altruistic acts can be conceptually distinguished from the motivations that underlie them. Research in evolutionary biology and evolutionary psychology suggests that such behaviors may also provide indirect benefits to the actor through mechanisms such as direct and indirect reciprocity, kin selection, network reciprocity, and group selection (Nowak, 2006), as well as costly signaling and mate selection (Miller, 2000, 2009).

Historically, much of the prosociality literature has focused on behaviors directed toward benefiting other humans. Indeed, in altruism research, “benefiting others” typically refers to interactions with partners, friends, acquaintances, or even strangers, at a personal cost (Eisenberg et al., 2015). Recent research, however, suggests that altruism is not constrained to human recipients. In particular, Otto et al. (2021) demonstrated that prosocial behavior can also extend to nature. From this perspective, prosocial propensity encompasses helping both people and the broader environment, with pro-environmental actions often considered a form of such helpful behavior (Neaman, 2025a, 2025b; Otto et al., 2021). In this sense, environmentalism can be viewed as a form of altruism, extending prosocial concern beyond humans to the planet itself (Duong and Pensini, 2023; Kaiser and Byrka, 2011; Kaiser et al., 2015; Klein et al., 2022; Nolan and Schultz, 2015). Consistent with this view, previous research has shown that altruism predicts helping behaviors toward both people and the environment, indicating a general tendency to benefit others (Gebauer et al., 2015; Tapia-Fonllem et al., 2013) as well as to care for nature (Clark et al., 2003; Tapia-Fonllem et al., 2013). Thus, individual differences in altruism may reflect a generalized propensity for prosocial action, regardless of whether the recipient is human or non-human.

Connectedness to nature (also known as “connectedness with nature” or “nature relatedness”) is an important and well-established construct in environmental psychology (Häyrinen and Pynnönen, 2020; Tam, 2013). It refers to the degree to which individuals perceive themselves as part of the natural world, encompassing affective, cognitive, and experiential bonds with nature (Mayer and Frantz, 2004; Nisbet et al., 2009). Notably, while its definition emphasizes both affective-emotional and cognitive aspects, the literature does not clearly agree on which dimensions are most central to the construct (Tiscareno-Osorno et al., 2023). As such, some authors have focused on the affective aspect, reflecting feelings toward nature (e.g., Brügger et al., 2011), whereas others emphasize the cognitive aspect (e.g., Schultz, 2002). Nevertheless, research on connectedness to nature generally suggests that it predicts pro-environmental actions (Guazzini et al., 2025; Restall and Conrad, 2015).

Building on the concept of emotional connectedness to nature, Neaman et al. (2025) defined “connectedness to soil” as an emotional bond that farmers develop with the soil. Although “connectedness to soil” may appear similar to “connectedness to nature”, Neaman et al. (2026) demonstrated its unique role. Specifically, that study employed the emotional connectedness to soil scale developed by Neaman et al. (2025) and the emotional connectedness to nature scale from Brügger et al. (2011), demonstrating their distinctiveness in a sample of Peruvian farmers. Furthermore, farmers’ pro-environmental propensity (operationalized as general ecological behavior) had a relatively large total effect on soil conservation behavior; however, when soil connectedness and nature connectedness were introduced as mediators in a parallel mediation model, full mediation was observed. Importantly, the indirect effect via soil connectedness was statistically significant, whereas the indirect effect via nature connectedness was not. This suggests that soil connectedness captures something distinct from, and more proximally relevant to, soil conservation behavior than general connectedness to nature.

Building on previous research showing that an individual’s prosocial propensity underpins their connectedness to nature (Neaman et al., 2023; Neaman et al., 2022; Otto et al., 2021), we posit here that prosocial propensity may also underpin connectedness to soil. The present study focused on farmers due to their everyday contact with soil, whereas the general population is typically more distant from it (Schaal et al., 2026). Although people may engage with soil in various ways beyond farming—such as gardening, growing house plants, or composting—prior studies on the general Chilean population indicate that such engagement is relatively infrequent (Díaz-Siefer et al., 2015; Neaman et al., 2021; Otto et al., 2021). This is further supported by Neaman et al. (2018), who found that even urban agronomy students engaged little in soil-related activities despite their academic background. Together, these findings align with the suggestion that urban residents may require “sensitization about soils” (Lal et al., 2021).

In the present study, the main objective is to evaluate the relationship between farmers’ prosocial propensity and their connectedness to soil. Specifically, the focus is on the cognitive aspects of connectedness to nature and connectedness to soil, given that the emotional aspects of these constructs were previously examined in the aforementioned study by Neaman et al. (2025). One of the most widely used measures of the cognitive dimension of connectedness to nature is the Inclusion of Nature in Self (INS) scale developed by Schultz (2002). Here, inclusion of nature in self refers to the extent to which an individual incorporates nature within their cognitive representation of the self. Supporting its validity, Brügger et al. (2011) found that the INS correlates with several other nature connectedness measures, including environmental identity (Clayton, 2003), connectedness to nature (Mayer and Frantz, 2004), implicit associations with nature (Schultz et al., 2004), and disposition to connect with nature, suggesting that it captures a meaningful and well-established dimension of the broader construct. Given its psychometric robustness and widespread use, the INS scale was adopted in the present study as the measure of cognitive connectedness to nature and, by extension, as the basis for operationalizing cognitive connectedness to soil as Inclusion of Soil in Self (ISS).

## Methods

### Participants

This study included 166 Chilean farmers (74% male) from the Valparaíso Region (central Chile) and the Arica and Parinacota Region (northern Chile). For clarity, the term “farmers” in this study refers to individuals responsible for making independent decisions related to farm soil management. This included the following groups: independent farmers (87%), agricultural company employees (6%), agricultural company owners (4%), and those who did not specify belonging to any of these three groups (3%), regardless of the size of their farm or their possession (or lack thereof) of a professional degree in agriculture. Notably, no statistically significant differences were present between these groups for the response variables of altruism, INS, and ISS (ANOVA, *p* > 0.05).

To determine the minimum sample size for this study, we considered effect sizes observed in previous Chilean research on the relationship between altruism (used as a measure of prosocial propensity) and connectedness to nature (Neaman et al., 2023; Neaman et al., 2022; Otto et al., 2021). This research indicated correlations in the range from *r* = 0.28 to *r* = 0.36. To detect a correlation of *r* = 0.28, with power = 0.90 and alpha = 0.05 (two-tailed), a minimum of 129 participants were required (Faul et al., 2007). With 166 participants, the study was able to detect small to moderate effects.

A surveyor traveled through agricultural areas in the aforementioned regions and visited individual farms. Farmers were invited to complete a paper-and-pencil questionnaire. No incentives were offered for participation. Prior to data collection, participants were informed about the purpose of the study and potential implications. This informed consent process emphasized the confidential, anonymous, and voluntary nature of participation, allowing participants to withdraw from the survey at any point. Data were collected in a manner that respected the participants’ time and privacy, in accordance with the ethical standards of research.

### Measures

Data were collected through self-report surveys that included the following three scales: (1) altruism (as an indicator of prosocial propensity), (2) connectedness to soil, and (3) connectedness to nature.

(1) In line with previous research in Chilean populations (Otto et al., 2021), altruism was used as the indicator of prosocial propensity and was measured using the altruism scale from Otto et al. (2021) in Spanish, adapted from the original self-report altruism scale by Rushton et al. (1981). This measure contains 26 items (Table 1) and uses a 5-point Likert scale ranging from (1) “never” to (5) “always.” Its convergent validity in Chilean samples has been previously established (Otto et al., 2021). Scale reliability and unidimensionality are discussed below.

**Table 1:** Altruism scale used in the study.

Code	Item	*δ*	MS
A12	I give a stranger a lift in my car.	2.93	1.05
A8	I donate blood.	2.73	0.98
A22	I put myself at risk to help someone I do not know.	2.18	0.95
A3	I change money for a stranger.	1.92	0.93
A18	I comfort a stranger who is crying.	1.62	0.88
A5	I give money to a stranger in need.	1.39	0.87
A7	I do volunteer work for a charity.	1.39	1.05
A24	When I see a stranger being mocked on the street, I defend them.	1.34	1.07
A25	I spend time with strangers who appear to be alone at an event.	1.23	0.94
A4	I give money to a charity or fundraising campaign.	0.92	0.99
A21	I am kind to strangers who seem unfriendly or rude at first.	0.88	1.00
A6	I donate goods or clothes to charity.	0.76	0.89
A20	*When a stranger asks me something, I do not listen and I continue on my way.*	0.26	1.24
A1	I help push a stranger’s broken car.	0.19	1.04
A11	I allow someone to go ahead of me in a line (driving a car, in the supermarket).	0.02	1.08
A14	I buy a “Teletón” product^*^.	−0.12	1.12
A9	I help carry a stranger’s belongings (bags, parcels, etc.).	−0.13	0.96
A10	I delay an elevator by holding the door for a stranger.	−0.14	0.95
A15	*I refuse to aid a stranger asking for help.*	−0.14	1.02
A17	I offer my seat on public transport to a stranger who is standing.	−0.66	1.02
A16	I offer to help elderly strangers cross the street.	−0.71	1.01
A19	I help an unknown person who has fallen on the street.	−0.79	1.02
A2	I give directions to a stranger.	−0.96	0.89
A23	*I do not care if my actions affect others.*	−1.46	1.16
A13	When I receive extra change, I give it back to the cashier.	−2.10	1.02
A26	If a stranger drops something, I pick it up and give it back to them.	−3.11	0.92

^*^In Chile, Teletón (a massive telethon) is a nationwide, annual charitable event.

Items in *italics* were negatively formulated and reversely coded before the analysis. These items should be read as “I refrain from…”. A 5-point Likert scale was used, offering nuanced responses with options including “never,” “rarely,” “sometimes,” “often,” and “always”. Item difficulties (*δ*) are presented in logits; larger logit values represent higher scores on each corresponding scale. MS is infit mean square, a fit statistic used in Rasch modeling to assess whether the responses to a given item conform to model predictions; values of MS ≤ 1.2 are considered good, while 1.2 < MS ≤ 1.3 are considered acceptable (Wright et al., 1994).

Importantly, to minimize direct reciprocity bias as a potential confound, the items in the altruism scale explicitly referred to actions toward strangers—that is, actions performed without the expectation of direct reciprocity. This distinguishes the construct from reciprocal altruism, which requires that individuals are able to identify one another and remember past interactions (Nowak, 2006). However, it should be noted that the items do not fully exclude indirect reciprocity, whereby engaging in altruistic behavior may still benefit the actor through reputational gains or by signaling cooperative intentions to third parties (Balliet et al., 2021).

**Figure 1:**
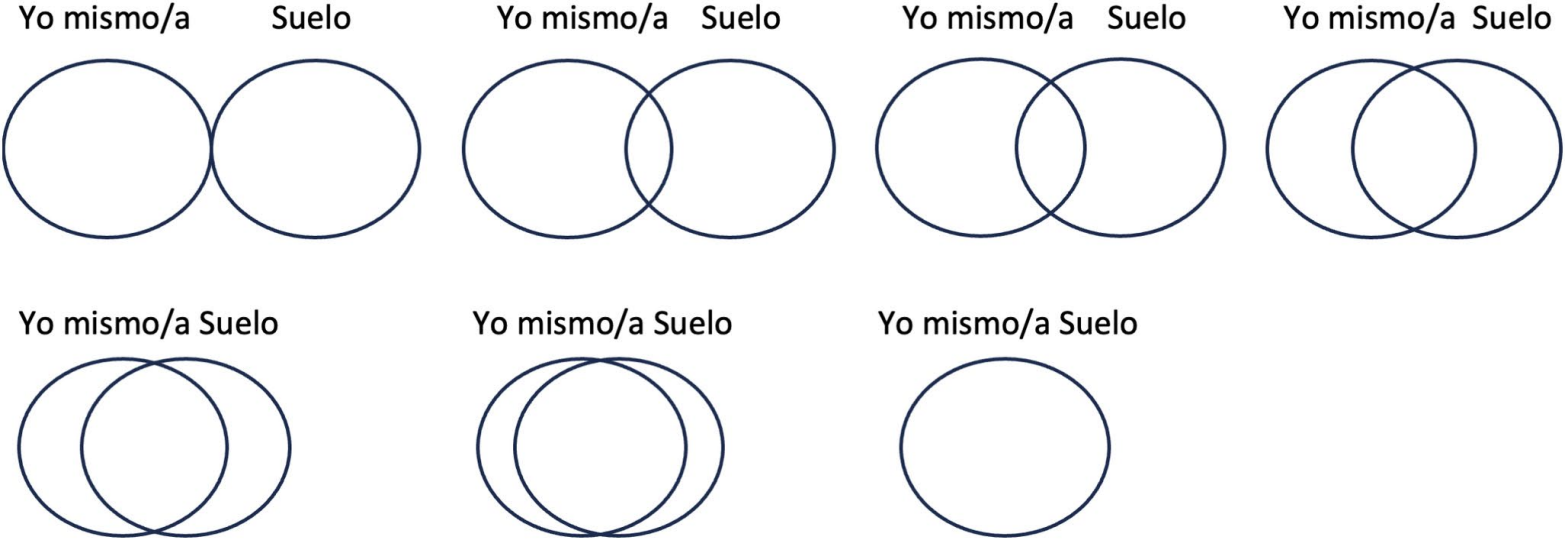
The new measure of inclusion of soil in self used in the study. In Spanish, “*Yo mismo/a*” means “Self,” whereas “*Suelo*” means “Soil.” The following instruction was given: “Please mark the image that best describes your relationship with the soil. The first option (separate circles) means that I and the soil are independent, while the last option (a single circle) means that I and the soil are one. Other options are intermediate, depending on the degree to which the circles overlap”.

(2) In this study, the Spanish version of the INS measure was used (Corraliza and Bethelmy, 2011). “Self” was translated to Spanish as “*Yo mismo/a*,” and “Nature” was translated to Spanish as “*Naturaleza*.”
(3) In this study, a new ISS measure is proposed, inspired by the INS measure developed by Schultz (2002), in which “Nature” is replaced by “Soil” (“*Suelo*” in Spanish, Figure 1).

Notably, two versions of the pen-and-pencil survey were used. In version A (*n* = 88), the order of the scales was as follows: INS, altruism, ISS, and socio-demographic questions. In version B (*n* = 88), the order of the scales was as follows: ISS, altruism, INS, and socio-demographic questions. There were no statistically significant differences between versions A and B in INS and ISS scores (*t*-test, *p* > 0.05).

### Data analysis

The dichotomous Rasch model was used to compute scores for the altruism scale using the TAM package in RStudio (R Core Team, 2021). To minimize measurement error, all items were converted to a dichotomous format prior to analysis (Kaiser and Wilson, 2000). Specifically, responses of “never,” “rarely,” and “sometimes” were recoded as 0, whereas “often” and “always” were recoded as 1.

Table 1 presents the values of infit mean square (MS), a fit statistic used in Rasch modeling to assess whether responses to a given item conform to model predictions. Values of MS ≤ 1.2 are considered good, while 1.2 < MS ≤ 1.3 are considered acceptable (Wright et al., 1994). To test the unidimensionality of each scale, a principal components analysis of the Rasch residuals was conducted (Brentani and Golia, 2007).

Importantly, in Rasch-type models, item parameters and person parameters are modeled separately (Bond and Fox, 2007). Table 1 presents item difficulties in logits, with larger logit values indicating higher scores on each corresponding scale. Based on participants’ Rasch scores (altruism scale) and sum scores (INS and ISS measures), Pearson correlations were then computed (Table 2).

**Table 2:** Zero-order Pearson correlations between the variables under study.

Variable	1	2	3	4	5
1. Age	—				
2. Education	−0.49^***^	—			
3. Income	−0.19^*^	0.22^**^	—		
4. Altruism	n.s.	n.s.	n.s.	—	
5. Connectedness to nature (INS)	n.s.	n.s.	n.s.	0.22^**^	—
6. Connectedness to soil (ISS)	0.23^**^	n.s.	−0.16^*^	0.24^**^	0.92^***^

INS, Inclusion of nature in self; ISS, inclusion of soil in self.

^*^p < 0.05, ^**^p < 0.01, ^***^p < 0.001, n.s., not statistically significant (*p* > 0.05).

Additionally, the relationships between altruism and connectedness measures were examined while controlling for age, gender, education level, and income level. Gender was coded as 1 for male and 2 for female, whereas education and income levels were treated as ordinal variables. Furthermore, to examine the effect of gender on variables under study, a *t*-test was conducted.

## Results

### Unidimensionality of altruism scale and sociodemographic descriptive results

Person separation reliability, estimated from the Rasch model, was 0.79 for the altruism scale, with values of 0.70 or higher considered satisfactory (Bond and Fox, 2007). All items exhibited good or acceptable fit to the Rasch model (Table 1). Furthermore, the variance explained by Rasch residuals was 10%, below the 15% threshold considered indicative of a secondary dimension (Fisher, 2007), supporting the unidimensionality of the scale. Together, these results indicate that the altruism scale performed reliably and measured a single underlying construct.

Participant age was 49 ± 15 (hereafter, mean ± standard deviation), with a range of 24–82 years old. Participants’ educational level was 3.4 ± 1.4 (on a scale from 1 to 7) and income level was 2.0 ± 0.56 (on a scale from 1 to 3). Older farmers tended to have lower educational and income levels but exhibited higher connectedness to soil (Table 2). The effect of gender on the variables under study was not statistically significant (*t*-test, *p* > 0.05).

### Correlational results

**Figure 2:**
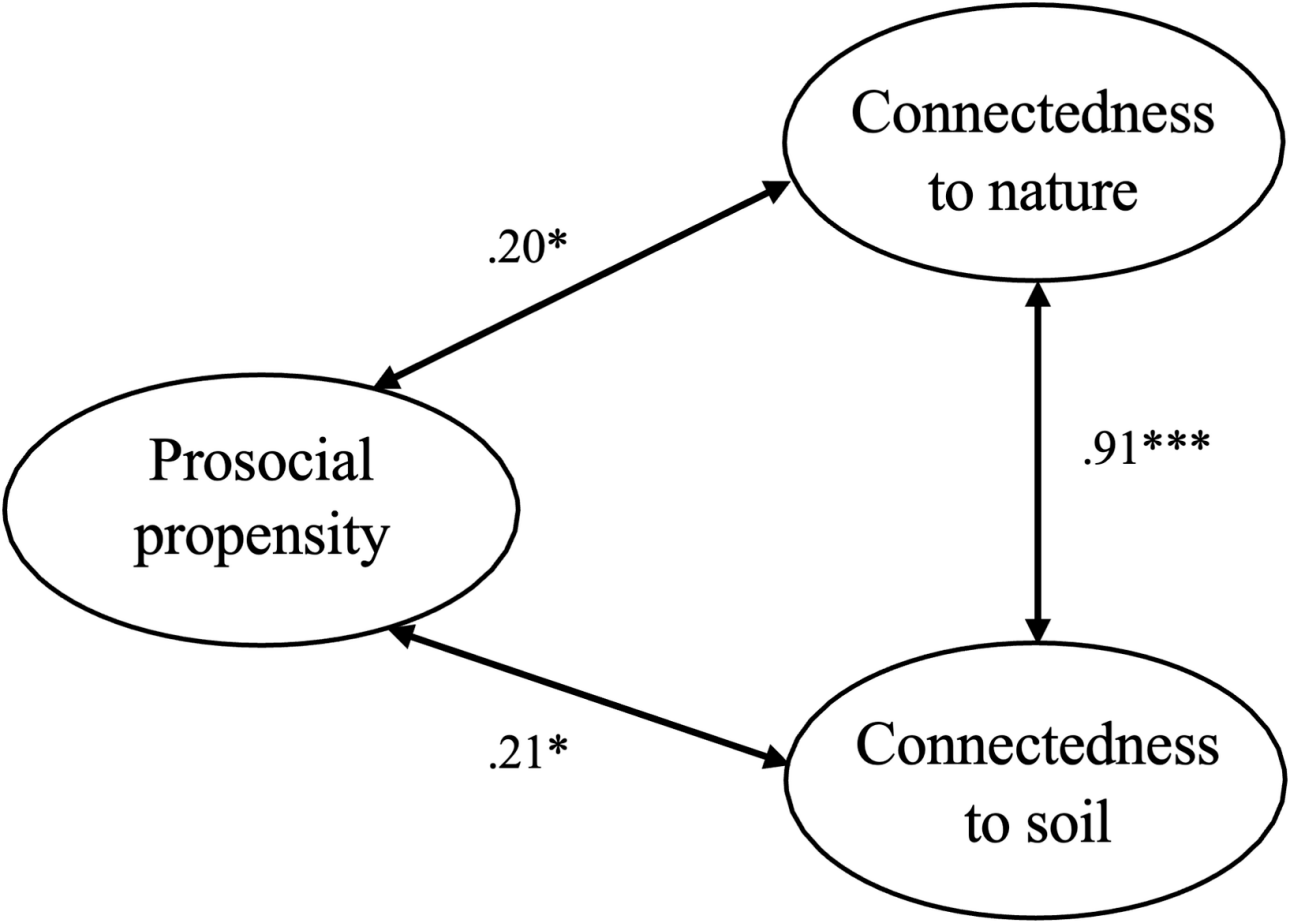
Schematic summary of key study findings. Relationships between altruism and connectedness measures, controlling for age, gender, education level, and income level. ^*^p < 0.01, ^***^p < 0.001.

The correlation between connectedness to nature (INS) and connectedness to soil (ISS) was positive and strong (*r* = 0.92, *p* < 0.001; Table 2). Positive correlations were also observed between altruism and connectedness to nature (*r* = 0.22, *p* < 0.01), and between altruism and connectedness to soil (*r* = 0.24, *p* < 0.01). Furthermore, when controlling for age, gender, education level, and income level, these relationships remained largely unchanged in terms of strength and statistical significance (Figure 2), suggesting that they are not driven by individual differences in sociodemographic characteristics.

## Discussion

The present study suggests that the cognitive aspects of connectedness to soil and connectedness to nature are closely related, as indicated by the strong correlation between INS and ISS scores. Notably, both scales capture only the cognitive dimension of the connection between people and nature (Häyrinen and Pynnönen, 2020; Tam, 2013) or soil. Future studies are warranted to compare scales reflecting different dimensions (cognitive versus affective) of these constructs in diverse farmer populations.

Beyond the relationship between the two connectedness scales, the correlation observed between altruism and connectedness to nature is consistent with previous Chilean research (Neaman et al., 2023; Neaman et al., 2022; Otto et al., 2021). This pattern is also supported by findings from Australia (Angelis and Pensini, 2023) and Türkiye (Yurtsever and Angin, 2022), collectively suggesting that the relationship between prosocial propensity and connectedness to nature is robust across different cultural contexts. Importantly, a similar relationship was observed between altruism and connectedness to soil, extending this pattern to a novel construct and suggesting that farmers’ prosocial propensity is associated not only with their general sense of connection to the natural environment, but also with the specific natural entity—soil—that is central to their daily work and livelihood.

The present results also align with Neaman et al. (2025) who found that older Chilean farmers tended to have lower educational and income levels but exhibited higher connectedness to soil. Similarly, the absence of gender effects observed here is consistent with Neaman et al. (2025) and with broader evidence suggesting no conclusive gender differences in either environmentalism (Klein et al., 2012; Larson et al., 2011) or prosociality (Monk-Turner et al., 2002; Neaman, 2025a).

### Limitations

While this study advances understanding of the relationship between environmentalism and prosociality, several limitations warrant consideration. The convenience sampling approach, concentrated in specific regions of Chile at a single time point, may limit the generalizability of findings. Future research should aim to replicate these results in broader and more diverse samples across different cultural and agricultural contexts.

In addition, the cross-sectional design precludes causal inference, and the single time point prevents examination of how the observed relationships develop over time. Nevertheless, given that connectedness to nature tends to be relatively stable in adults (Hughes et al., 2019), cross-sectional designs may still provide meaningful insights into the relationships examined here.

### Theoretical value of the study

A novel contribution of this study is the demonstration that prosocial propensity is positively related to connectedness to both nature and soil. Importantly, this finding extends the growing body of evidence linking environmentalism and prosociality in a Chilean farming population. This context is both culturally and occupationally distinct from the predominantly Western, urban, and student samples that dominate the environmental psychology literature (Tam and Milfont, 2020).

Moreover, this study provides the first evidence that connectedness to soil may reflect a domain in which prosocial tendencies are expressed. In addition, it is among the few studies to compare connectedness to nature and connectedness to soil in populations with a potentially more meaningful relationship to soil, namely, farmer populations.

## Data Availability

The raw data supporting the conclusions of this article will be made available by the authors, without undue reservation.
